# Diagnosis of an Umbilical Vein Aneurysm at 30 Weeks Gestation

**DOI:** 10.1155/2021/2433252

**Published:** 2021-11-16

**Authors:** Meredith S. Campbell, Amanda M. Craig, Jeff Reese, Alicia K. Crum, Soha S. Patel

**Affiliations:** ^1^Division of Neonatology, Department of Pediatrics, Monroe Carell Jr. Children's Hospital at Vanderbilt, Nashville, TN, USA; ^2^Department of Obstetrics and Gynecology, Vanderbilt University Medical Center, Nashville, TN, USA; ^3^Division of Maternal-Fetal Medicine, Department of Obstetrics and Gynecology, Vanderbilt University Medical Center, Nashville, TN, USA

## Abstract

**Background:**

Fetal umbilical vein aneurysm is an uncommon anomaly without clear guidelines regarding the management of these pregnancies. *Case Presentation*. We describe an ultrasound diagnosis of this condition involving a 38-year-old multigravid woman who presented at 30 weeks and 3 days gestation with severe fetal growth restriction, reverse end-diastolic flow on umbilical artery dopplers, elevated ductus venosus doppler, and an umbilical vein aneurysm. Due to nonreassuring fetal assessment in the setting of an umbilical vein aneurysm, she underwent a cesarean delivery with a favorable neonatal outcome.

**Conclusions:**

There are currently no guidelines for the management of an umbilical vein aneurysm. This case demonstrates a successful multidisciplinary approach for creating a plan of care focused on achieving a favorable outcome for a fetus with a large umbilical vein aneurysm. The approach took into account timing of delivery given the potential for fetal morbidity and mortality, while factoring in the risk of prematurity.

## 1. Introduction

An umbilical vein aneurysm is a rare prenatal diagnosis with limited literature available regarding management. Fetal umbilical vein aneurysm was first described in 1978 [1] and accounts for less than 4% of umbilical cord malformations [2, 3]. Possible complications include aneurysm rupture, thrombosis, and compression of the umbilical cord leading to poor fetal outcome [4]. Published studies have been small, with contradictory results regarding increased risk of fetal anomalies or poor perinatal outcomes associated with this ultrasonographic finding. Some authors have reported normal fetal outcomes [4, 5] while other authors have cited fetal mortality rates over 40% [6, 7]. A review of 23 cases identified no fetal or neonatal deaths; however, these authors did find a high rate of fetal malformations with 34.8% of infants having major congenital anomalies including multiple-anomaly syndromes and severe cardiac defects [8].

Our case highlights a multidisciplinary approach to the care of a woman at 30 weeks gestation whose pregnancy was complicated by this abnormality. We recommend that obstetrical providers consider earlier timing of delivery in the setting of umbilical vein aneurysm due to the potential for severe fetal morbidity and mortality.

## 2. Case Presentation

### 2.1. Maternal History

A 38-year old gravida 2, para 1001 mother presented at 30 weeks and 3 days gestation as a transfer of care from an outside hospital for reverse end-diastolic flow on umbilical artery Doppler assessment, elevated ductus venosus doppler, and umbilical vein aneurysm in the setting of severe fetal growth restriction, less than the 3^rd^ percentile for gestational age. Her pregnancy was also complicated by type 2 diabetes mellitus, a prior history of cesarean delivery, and an increased risk for trisomy 21 (1 : 80) on first trimester screen. Prior to transfer to our hospital, she received two doses of betamethasone and a magnesium sulfate infusion for fetal neuroprotection.

**Figure 1:**
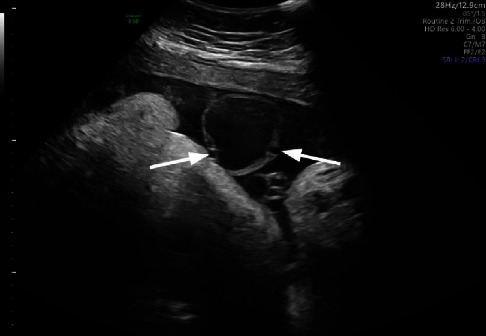
Cross-sectional view of the umbilical vein aneurysm with arrows pointing towards the umbilical arteries on either side.

She was admitted to the hospital for observation and continuous electronic fetal monitoring. Repeat ultrasound on arrival confirmed the abnormal Doppler findings. Elevated flow through the ductus venosus with intermittent reversal of end-diastolic flow. More concerning, there was a 3-4 cm thin-walled umbilical vein aneurysm in the free-floating cord with nonpulsatile, turbulent flow (Figure 1). The following day, given her ultrasound findings, and increased risk for fetal compromise, the decision was made to schedule a repeat cesarean section. The surgery was tolerated well with no complications. Postoperatively, she was diagnosed with HELLP syndrome (hemolysis, elevated liver enzymes, low platelet count) and started on magnesium sulfate for seizure prophylaxis. She was subsequently discharged on postoperative day 3.

### 2.2. Birth History and Neonatal Evaluation

A 1320 gram male infant, born at less than the 1^st^ percentile for weight, was delivered with APGAR scores of 6 and 7 at 1 and 5 minutes, respectively. Resuscitation included stimulation and continuous positive airway pressure (CPAP). The infant was admitted to the neonatal intensive care unit for management of prematurity and respiratory distress.

Laboratory tests revealed a hemoglobin of 15.9 gm/dL, platelet count 23 × 103/mcL, requiring platelet transfusion. Neonatal thrombocytopenia was considered secondary to maternal HELLP syndrome and prematurity, although a relationship to the umbilical cord anomaly such as thrombus formation within the umbilical aneurysm was also considered. Abdominal ultrasound was performed to evaluate secondary causes of thrombocytopenia including sequestration at the umbilical cord site, arteriovenous malformation, or vascular lesions, which was negative. There were no signs of intracranial or intraventricular bleeding on ultrasound. Further studies including urine cytomegalovirus, karyotype, and chromosomal microarray analysis were negative.

**Figure 2:**
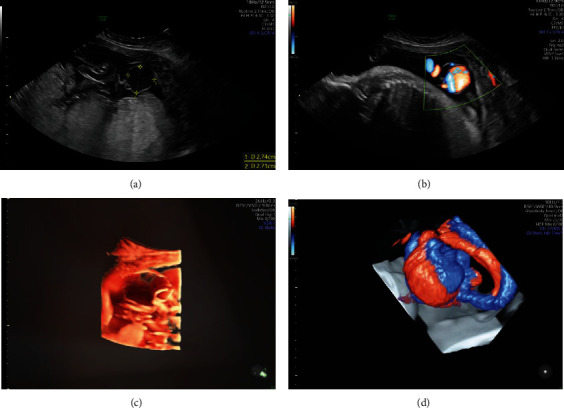
(a) Top left—2.7 cm umbilical vein aneurysm free-floating cord. (b) Top right—color doppler demonstrating bidirectional flow within umbilical vein aneurysm. (c) Bottom left—high definition live 3D rendering of umbilical vein aneurysm. (d) Bottom right—color 3D rendering of umbilical vein aneurysm.

**Figure 3:**
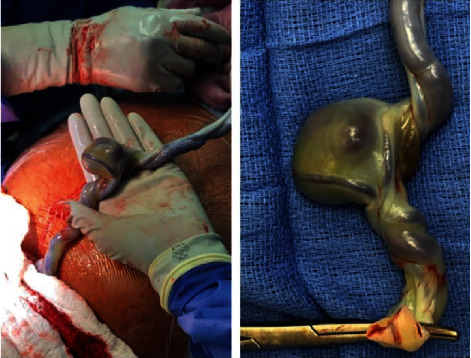
Gross specimen of the umbilical cord and umbilical cord aneurysm.

The extra-abdominal umbilical vein aneurysm measured approximately 3.5 × 3 cm (Figures 2 and 3). Placental pathology showed uneven villous maturation, fetal thrombotic vasculopathy with endothelial cushion lesions, hemorrhagic endovasculitis, and focal acute deciduitis. The placenta was grossly normal with a normal placental cord insertion.

Initial chest X-ray showed decreased lung volumes, diffuse granularity, and air bronchograms most consistent with respiratory distress syndrome. The infant required intubation, a single dose of surfactant, and mechanical ventilation for 1 day. After extubation, he remained on nasal CPAP until 34 weeks gestation followed by low flow nasal cannula until 37 weeks gestation, consistent with mild bronchopulmonary dysplasia. The infant was discharged on day of life 67.

## 3. Discussion and Conclusion

Recommendations regarding evaluation and antenatal surveillance are not well established but are aimed at recognizing early evidence of fetal distress to potentially prevent poor fetal outcomes. Authors from a case report of an intrauterine fetal demise at 41 weeks gestation have suggested that the increased umbilical cord blood flow in pregnancy may increase fetal compromise with advancing gestational age, which might explain why cases of intrauterine death have been presented later in pregnancy [9]. These authors hypothesized that there is a significantly increased risk of vessel rupture with uterine contractions in labor and rupture of membranes [9]. Due to the uncertainties regarding the relationship of this hemodynamically significant lesion with poor neonatal outcomes, obstetric providers should consider earlier timing of delivery after administration of betamethasone given the potential for severe fetal morbidity and mortality [10–13].

## Data Availability

Data sharing is not applicable to this article as no datasets were generated or analyzed during the current study.

## Abbreviations

CPAP: Continuous positive airway pressure
HELLP syndrome: Hemolysis, elevated liver enzymes, low platelet count.
